# The McNemar test for binary matched-pairs data: mid-*p* and asymptotic are better than exact conditional

**DOI:** 10.1186/1471-2288-13-91

**Published:** 2013-07-13

**Authors:** Morten W Fagerland, Stian Lydersen, Petter Laake

**Affiliations:** 1 Unit of Biostatistics and Epidemiology, Oslo University Hospital, Oslo, Norway; 2 Regional Centre for Child and Youth Mental Health and Child Welfare-Central Norway, Norwegian University of Science and Technology, Trondheim, Norway; 3 Department of Biostatistics, University of Oslo, Oslo, Norway

**Keywords:** Matched pairs, Dependent proportions, Paired proportions, Quasi-exact

## Abstract

**Background:**

Statistical methods that use the mid-*p* approach are useful tools to analyze categorical data, particularly for small and moderate sample sizes. Mid-*p* tests strike a balance between overly conservative exact methods and asymptotic methods that frequently violate the nominal level. Here, we examine a mid-*p* version of the McNemar exact conditional test for the analysis of paired binomial proportions.

**Methods:**

We compare the type I error rates and power of the mid-*p* test with those of the asymptotic McNemar test (with and without continuity correction), the McNemar exact conditional test, and an exact unconditional test using complete enumeration. We show how the mid-*p* test can be calculated using eight standard software packages, including Excel.

**Results:**

The mid-*p* test performs well compared with the asymptotic, asymptotic with continuity correction, and exact conditional tests, and almost as good as the vastly more complex exact unconditional test. Even though the mid-*p* test does not guarantee preservation of the significance level, it did not violate the nominal level in any of the 9595 scenarios considered in this article. It was almost as powerful as the asymptotic test. The exact conditional test and the asymptotic test with continuity correction did not perform well for any of the considered scenarios.

**Conclusions:**

The easy-to-calculate mid-*p* test is an excellent alternative to the complex exact unconditional test. Both can be recommended for use in any situation. We also recommend the asymptotic test if small but frequent violations of the nominal level is acceptable.

## Background

Matched-pairs data arise from study designs such as matched and crossover clinical trials, matched cohort studies, and matched case-control studies. The statistical analysis of matched-pairs studies must make allowance for the dependency in the data introduced by the matching. A simple and frequently used test for binary matched-pairs data is the McNemar test. Several versions of this test exist, including the asymptotic and exact (conditional) tests. The traditional advice is to use the asymptotic test in large samples and the exact test in small samples. The argument for using the exact test is that the asymptotic test may violate the nominal significance level for small sample sizes because the required asymptotics do not hold. One disadvantage with the exact test is conservatism: it produces unnecessary large *p*-values and has poor power.

Consider the data in Table 1, which gives the results from a study by Bentur et al. [1]. Airway hyper-responsiveness (AHR) status—an indication of pulmonary complications—was measured in 21 children before and after stem cell transplantation (SCT). The incidence of AHR increased from two (9.5%) children before SCT to eight (38%) children after SCT. The asymptotic test gives *p*=0.034, and the exact test gives *p*=0.070. The two *p*-values are considerably different, which often happens when we have a small sample size. The next example shows that this may also be the case for large sample sizes.

**Table 1:** **Airway hyper-responsiveness (AHR) status before and after stem cell transplantation (SCT) in 21 children **[1]

		**After SCT**		
		**AHR**	**No AHR**	**Sum**
Before SCT	AHR	1	1	2
	No AHR	7	12	19
	Sum	8	13	21

In another study of SCT, 161 myeloma patients received consolidation therapy three months after SCT [2]. Complete response (CR) was measured before and after consolidation (Table 2). An increase in CR following consolidation was observed: sixty-five (40%) patients had CR before consolidation compared with 75 (47%) patients after consolidation. The asymptotic test gives *p*=0.033, and the exact test gives *p*=0.053.

**Table 2:** **Complete response (CR) before and after consolidation therapy **[2]

		**After consolidation**		
		**CR**	**No CR**	**Sum**
Before consolidation	CR	59	6	65
	No CR	16	80	96
	Sum	75	86	161

The choice between an asymptotic method and a conservative exact method—which can be summarized as a trade-off between power and preservation of the significance level—is well known from other situations involving proportions [3]. For the independent 2×2 table, a good compromise can be reached using the mid-*p* approach [4]. The Fisher mid-*p* test, which is a modification of Fisher’s exact test, combines excellent power with rare and minor violations of the significance level [5]. The modification required to transform an exact *p*-value to a mid-*p*-value is simple: the mid-*p*-value equals the exact *p*-value minus half the point probability of the observed test statistic.

The purpose of this article is to investigate whether a mid-*p* version of the McNemar exact conditional test can offer a similar improvement for the comparison of matched pairs as has been observed with independent proportions. A supplementary materials document (Additional file 1) shows how the mid-*p* test can be calculated using several standard software packages, including Excel, SAS, SPSS, and Stata.

## Methods

### 

#### Notation

Let *N* denote the observed number of matched pairs of binomial events A and B—where the possible outcomes are referred to as success (1) or failure (2)—and let (*Y*_*i*1_,*Y*_*i*2_) denote the outcome of the *i*th pair. The observed data may be summarized in a 2×2 contingency table, as in Table 3. Each *n*_*kl*_ for *k*,*l*=1,2 corresponds to the number of event pairs (*Y*_*i*1_,*Y*_*i*2_) with outcomes *Y*_*i*1_=*k* and *Y*_*i*2_=*l*. Let *p*_*kl*_ denote the joint probability that *Y*_*i*1_=*k* and *Y*_*i*2_=*l*, which we assume independent of *i*. Following the notation in Agresti [6, pp. 418–420], this is a marginal or a population-averaged model. We denote the probabilities of success for events A and B—or equivalently, the marginal probabilities that *Y*_*i*1_=1 and *Y*_*i*2_=1—by *p*_1+_ and *p*_+1_, respectively. The null hypothesis of interest is *H*_0_: *p*_+_=*p*_+1_. The alternative hypothesis is *H*_1_: *p*_1+_≠*p*_+1_.

**Table 3:** **The observed counts (and joint outcome probabilities) of a paired *2 × 2 *table**

		**Event B**		
		**Success**	**Failure**	**Sum**
Event A	Success	*n*_11_(*p*_11_)	*n*_12_(*p*_12_)	*n*_1+_(*p*_1+_)
	Failure	*n*_21_(*p*_21_)	*n*_22_(*p*_22_)	*n*_2+_(*p*_2+_)
	Sum	*n*_+1_(*p*_+1_)	*n*_+2_(*p*_+2_)	*N* (1)

It might, however, be more realistic to assume that *p*_*kl*_also depends on the subject *i*. As denoted by Agresti [6, pp. 418–420], this is a subject-specific model. Further, this is a conditional model, since we are interested in the association within the pair, conditioned on the subject. Data from *N* matched pairs are then presented in *N* 2×2 tables, one for each pair. Collapsing over the pairs results in Table 3. Conditional independence between *Y*_1_ and *Y*_2_ is tested by the Mantel-Haenszel statistic [6, p.417]. But that test statistic is algebraically equal to the squared McNemar test statistic. In the following, we will not specify whether we test for marginal homogeneity or conditional independence.

#### The asymptotic McNemar test

The asymptotic McNemar test conditions on the number of discordant pairs (*n*_12_+*n*_21_). Conditionally, *n*_12_ is binomially distributed with parameters *n*=*n*_12_+*n*_21_ and *p*=1/2 under the null hypothesis. The asymptotic McNemar test statistic [7], which is the score statistic for testing marginal homogeneity, is

(1) $$z=\frac{n_{12}-n_{21}}{\sqrt{n_{12}+n_{21}}},$$

and its asymptotic distribution is the standard normal distribution. The equivalent McNemar test statistic *χ*^2^=*z*^2^=(*n*_12_−*n*_21_)^2^/(*n*_12_+*n*_21_) is approximately chi-squared distributed with one degree of freedom under the null hypothesis. The asymptotic McNemar test is undefined when *n*_12_=*n*_21_=0.

#### The asymptotic McNemar test with continuity correction

Edwards [8] proposed the following continuity corrected version of the asymptotic McNemar test:

(2) $$z=\frac{|n_{12}-n_{21}|-1}{\sqrt{n_{12}+n_{21}}}.$$

The asymptotic McNemar test with continuity correction (CC) approximates the exact conditional test. Hence, it combines the disadvantage of an asymptotic test (significance level violations) with the disadvantage of a conditional exact test (overly conservativeness), and we do not expect it to perform well. We include it in our evaluations because it features in influential textbooks such as Altman [9] and Fleiss et al. [10]. The asymptotic McNemar test with continuity correction is undefined when *n*_12_=*n*_21_=0.

#### The McNemar exact conditional test

The test statistic in (1) measures the strength of the evidence against the null hypothesis. If we, as in the derivation of the asymptotic test, condition on the number of discordant pairs (*n*_12_+*n*_**21**_), we can use the simple test statistic *n*_12_ to derive an exact conditional test. The conditional probability under *H*_0_ of observing any outcome *x*_12_ given *n*=*n*_12_+*n*_21_ discordant pairs is the binomial point probability

(3) $$f(x_{12}|n)=\left(\;\;\begin{array}{l} n\\ x_{12} \end{array}\;\;\right){\left(\frac{1}{2}\right)}^{n}.$$

The McNemar exact conditional one-sided *p*-value is obtained as a sum of probabilities:

(4) $$\text{one-sided}\;\;p\text{-value}=\overset{\min(n_{12},n_{21})}{\underset{x_{12}=0}{\sum }}f(x_{12}|n),$$

and the two-sided *p*-value equals twice the one-sided *p*-value. If *n*_12_=(*n*_12_+*n*_21_)/2, the *p*-value equals 1.0. The exact conditional test is guaranteed to have type I error rates not exceeding the nominal level.

#### The McNemar mid-*p* test

A mid-*p*-value is obtained by first subtracting half the point probability of the observed *n*_12_ from the exact one-sided *p*-value, then double it to obtain the two-sided mid-*p*-value [4]. Hence, the McNemar mid-*p*-value equals

(5) $$\begin{array}{lc} \text{mid-}p\text{-value}=2\cdot \left[\text{one-sided}\;\;p\text{-value}-\frac{1}{2}f(n_{12}|n)\right]\\ \;=\text{two-sided}\;\;p\text{-value}-f(n_{12}|n), & \end{array}$$

where *f* is the probability function in (3). If *n*_12_=*n*_21_, substitute (5) with

(6) $$\text{mid-}p\text{-value}=1-\frac{1}{2}f(n_{12}|n).$$

The type I error rates of the mid-*p* test—as opposed to those of exact tests—are not bounded by the nominal level; however, in a wide range of designs and models, both mid-*p* tests and confidence intervals violate the nominal level rarely and with low degrees of infringement [11-13]. Because mid-*p* tests are based on exact distributions, they are sometimes called quasi-exact [14]. Additional file 1 provides details on how to calculate the McNemar mid-*p* test with several standard software packages.

#### An exact unconditional test

The tests in the previous sections did not used the concordant pairs of observations (*n*_11_ and *n*_22_) in their calculations. The unconditional approach is to consider all possible tables with *N* pairs and thereby use information from all observed pairs, including the concordant ones. The exact unconditional test attributed to Suissa and Shuster [15] uses the McNemar test statistic (1). Let *z*_obs_ be the observed value, and let

(7) $$z(x)=\frac{x_{12}-x_{21}}{\sqrt{x_{12}+x_{21}}},$$

where **x**=(*x*_11_,*x*_12_,*x*_21_,*x*_22_) denotes a possible outcome with *N* pairs, and let *n*=*x*_12_+*x*_21_. If, for a one-sided test, *z*_obs_≥0, the potential outcomes that provide at least as much evidence against the null hypothesis as the observed outcome—namely those with *z*(**x**)≥*z*_obs_—are the pairs (*x*_12_,*n*) in the region

(8) $$\;C\;=\;\left\{(x_{12},n)\;:x_{12}\;\geq \;h(n);\;x_{12}\;=\;0,1,\ldots ,n;\;n=0,1,\ldots ,N\right\}\;,$$

where *h*(*n*)=0.5·(*z*_obs_*n*^1/2^+*n*). Under the null hypothesis, the triplets (*x*_12_,*n*,*N*−*n*) are trinomially distributed with parameters *N* and (*p*/2,*p*/2,1−*p*), and the attained significance level is

(9) $$P(p)=\underset{C}{\sum }\left(\;\;\begin{array}{lll} N\\ x_{12} & n-x_{12} & N-n \end{array}\;\;\right){\left(\frac{p}{2}\right)}^{n}{\left(1-p\right)}^{N-n},$$

where *p* is the probability of a discordant pair (a nuisance parameter). We eliminate the nuisance parameter by maximizing *P*(*p*) over the range of *p*. After simplifying (9), we get the following expression for the exact unconditional one-sided *p*-value [15]:

(10) $$\begin{array}{c} \;\text{one-sided}\;\;p\text{-value}=\underset{0<p<1}{\sup}\left[\overset{N}{\underset{n=k}{\sum }}\left(\;\;\begin{array}{l} N\\ n \end{array}\;\;\right)p^{n}{\left(1-p\right)}^{N-n}\right)\\ \;\;\;\;\;\;\left(F_{n}(n-i_{n}-1)\right], \end{array}$$

where $k=\text{int}(z_{\text{obs}}^{2}+1)$, *F*_*n*_ is the cumulative binomial distribution function with parameters (*n*,1/2), *i*_*n*_=int{*h*(*n*)}, and int is the integer function. Suissa and Shuster [15] outline a numerical algorithm to find the supremum in (10). If *z*_obs_<0, the one-sided *p*-value is found by reversing the inequality in (8). The two-sided *p*-value equals twice the one-sided *p*-value.

#### Evaluation of the tests

To compare the performances of the five tests, we carried out an evaluation study of type I error rates and power. We used complete enumeration (rather than stochastic simulations) and a large set of scenarios. Each scenario is characterized by fixed values of *N* (the number of matched pairs), *p*_1+_ and *p*_+1_ (the probabilities of success for each event), and *θ*=*p*_11_*p*_22_/*p*_12_*p*_21_. *θ* can be interpreted as the ratio of the odds for the event *Y*_2_ given *Y*_1_. We use *θ* as a convenient way to re-parameterize {*p*_11_,*p*_12_,*p*_21_,*p*_22_} into {*p*_1+_,*p*_+1_,*θ*}, which includes the parameter of interest, namely the two marginal success probabilities. We used StatXact PROCs for SAS (Cytel Inc.) to calculate *p*-values of the exact unconditional test and Matlab R2011b (Mathworks Inc.) to calculate *p*-values of the four other tests and to perform the evaluation study. In cases where *n*_12_=*n*_21_=0, we set *p*=1 for the two asymptotic McNemar tests.

For the calculations of type I error rates, we used 19 values of *N* (10, 15, 20, …, 100), five values of *θ* (1.0, 2.0, 3.0, 5.0, 10.0), and 101 values of *p*_1+_=*p*_+1_ (0.00, 0.01, 0.02, …, 1.00), a total of 9595 scenarios. The nominal significance level was 5%.

Power was calculated for *N*=1, 2, …, 100, *θ*=1.0, 2.0, 3.0, 5.0, 10.0, *p*_1+_=0.1, 0.35, 0.6, and *Δ*=*p*_+1_−*p*_1+_=0.10, 0.15, 0.20, 0.25, 0.30, 0.35.

## Results

### 

#### Type I error rates

**Figure 1:**
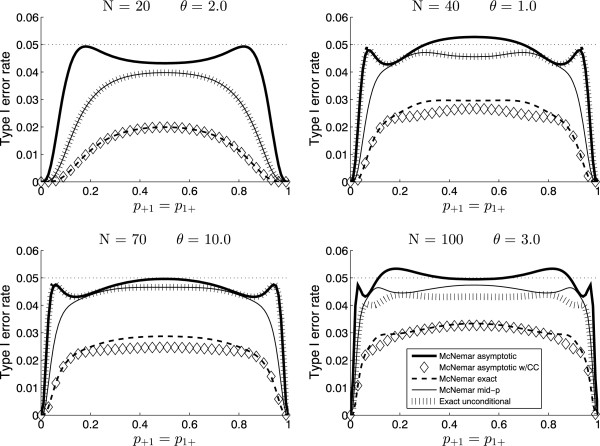
**Type I error rates as functions of the probability of success.** In each panel, the number of matched pairs (*N*) and the odds ratio (*θ*) are fixed.

The between tests differences in type I error rates were largely consistent across the considered scenarios. Figure 1 illustrates these differences. The type I error rates of the McNemar exact conditional test are low and barely above 3%, even for as much as 100 matched pairs. The asymptotic McNemar test with CC performs similarly to the exact conditional test but is even more conservative. The asymptotic McNemar test (without CC) has type I error rates close to the nominal level for most combinations of parameters. It violates the level quite often, although not by much. The exact unconditional and the McNemar mid-*p* tests perform similarly. For most combinations of parameters, the type I error rates of the two tests are identical. For some situations with small proportions, however, the exact unconditional test has type I error rates closer to the nominal level than does the mid-*p* test (Figure 1, upper right and lower left panels). On the other hand, the mid-*p* test sometimes has type I error rates closer to the nominal level than does the exact unconditional test (Figure 1, lower right panel). Both tests are clearly superior to the McNemar exact conditional test and the asymptotic McNemar test with CC.

Table 4 presents summary statistics of the calculations of type I error rates. The mean and maximum type I error rate are shown for each test over all scenarios and for subregions based on the number of matched pairs. We also show the proportion of scenarios where the nominal significance level is violated and the proportion of scenarios where the type I error rate is below 3%. The asymptotic McNemar test violates the nominal significance level in 29% of the total number of considered scenarios. We note that this proportion is only 3.7% for small sample sizes (10≤*N*≤30) and as much as 52% for large sample sizes (65≤*N*≤100). A mitigating feature is that—as indicated in Figure 1—the infringement on the nominal significance level is small: the maximum type I error rate of the asymptotic McNemar test is 5.37%. If we are concerned with aligning the mean (instead of the maximum) type I error rate with the nominal level, the results in Table 4 suggest that the asymptotic McNemar test is the superior test, both overall and in each of the subregions based on sample size.

**Table 4:** Evaluation of type I error rates (TIER)

	**mean**	**max**	**proportion**	**proportion**
**Method**	**TIER**	**TIER**	**TIER*> 0.05***	**TIER*< 0.03***
All 9595 scenarios				
McNemar asymptotic	0.0430	0.0537	0.294	0.121
McNemar asymptotic w/CC	0.0190	0.0357	0.000	0.889
McNemar exact	0.0201	0.0367	0.000	0.880
McNemar mid-*p*	0.0349	0.0495	0.000	0.260
Exact unconditional	0.0373	0.0495	0.000	0.201
Subregion: 10≤*N*≤30(2525 scenarios)				
McNemar asymptotic	0.0352	0.0529	0.037	0.281
McNemar asymptotic w/CC	0.0089	0.0237	0.000	1.000
McNemar exact	0.0090	0.0278	0.000	1.000
McNemar mid-*p*	0.0212	0.0469	0.000	0.627
Exact unconditional	0.0251	0.0488	0.000	0.541
Subregion: 35≤*N*≤60(3030 scenarios)				
McNemar asymptotic	0.0435	0.0537	0.210	0.084
McNemar asymptotic w/CC	0.0196	0.0306	0.000	0.991
McNemar exact	0.0210	0.0306	0.000	0.989
McNemar mid-*p*	0.0374	0.0474	0.000	0.176
Exact unconditional	0.0408	0.0482	0.000	0.096
Subregion: 65≤*N*≤100(4040 scenarios)				
McNemar asymptotic	0.0476	0.0535	0.519	0.049
McNemar asymptotic w/CC	0.0249	0.0357	0.000	0.743
McNemar exact	0.0263	0.0367	0.000	0.723
McNemar mid-*p*	0.0416	0.0495	0.000	0.095
Exact unconditional	0.0423	0.0495	0.000	0.066

As expected, the two exact tests do not violate the nominal significance level in any of the considered scenarios. Interestingly, neither does the McNemar mid-*p* test.

Finally, one important comment to the interpretation of Table 4. The values of the parameters *p*_1+_ and *p*_+1_ were selected to represent the entire range of possible values and not to be a representative sample of the situations that might be encountered in practice. Scenarios with probabilities close to zero or one are thereby given more weight to the summary statistics in Table 4 than their impact in actual studies. Thus, the mean type I error rates of a typical study are likely closer to the nominal level than indicated in Table 4. The table is, however, a good illustration of the differences in performance between the five tests.

Further details of the results from the evaluation of type I error rates can be found in a supplementary materials document (Additional file 2), which contains box-plots of type I error rates from the total and various subregions of the evaluation study.

#### Power

**Figure 2:**
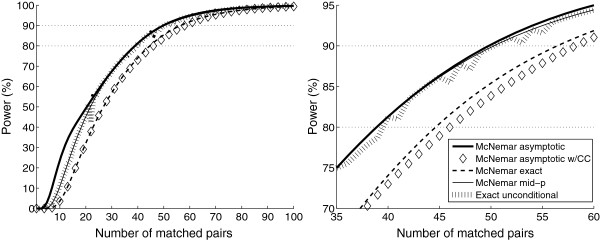
**Power of the tests as functions of the number of matched pairs.** The success probabilities (*p*_1+_= 0.1 and *p*_+1_= 0.35) and the odds ratio (*θ*=2.0) are fixed. The plot in the right panel shows details from the plot in the left panel.

Figure 2 shows the power of the tests as functions of the number of matched pairs with the usual yardsticks of 80% and 90% power marked in for reference. Only one combination of *p*_1+_, *p*_+1_, and *θ* is shown, however, the results where qualitatively equal for other settings. The powers of the asymptotic McNemar, the McNemar mid-*p*, and the exact unconditional tests are quite similar, although the asymptotic test is slightly better than the other two tests. The powers of the exact conditional test and the asymptotic McNemar test with CC trail that of the other tests considerably.

Table 5 displays the number of matched pairs needed to reach power of 50%, 60%, 70%, 80%, and 90% averaged over the 15 combinations of *θ*=1.0,2.0,3.0,5.0,10.0 and *p*_1+_=0.1,0.35,0.60. We show results for three of the *Δ*-values and note that similar results were obtained with *Δ*=0.1, 0.2, and 0.3. Values of *N* greater than 100 were estimated by simple linear extrapolation. The increase in sample size of using the exact unconditional or the mid-*p* test instead of the asymptotic McNemar test is quite small and in the range 0–3. The exact conditional test and the asymptotic McNemar test with CC, on the other hand, need a considerably greater sample size than the other tests. We emphasize that Table 5 is averaged over several combinations of parameters, and the values in it should not be used to plan the sample size of a study. The power of the tests are heavily dependent on the parameter values, even though the between tests differences in power were consistent across the different parameters in this evaluation. Table 5 thus illustrates typical sample size differences of the tests and not the actual sample size needed for a study.

**Table 5:** **The number of matched-pairs (*N*) needed to reach power of 50%, 60%, 70%, 80%, and 90%, averaged over five values of *θ * and three values of*p***_***1+***_**, for three values of*Δ=p***_***+1***_***−p***_***1+***_

	***N*to reach power of**				
	**50%**	**60%**	**70%**	**80%**	**90%**
*Δ*=0.15					
McNemar asymptotic	56	69	85	103^∗^	123^∗^
McNemar asymptotic w/CC	68	82	98	114^∗^	129^∗^
McNemar exact	67	81	96	113^∗^	129^∗^
McNemar mid-*p*	57	71	87	104^∗^	123^∗^
Exact unconditional	57	71	88	106^∗^	126^∗^
*Δ*=0.25					
McNemar asymptotic	23	28	34	42	54
McNemar asymptotic w/CC	30	35	41	50	63
McNemar exact	29	34	41	49	62
McNemar mid-*p*	24	29	35	43	56
Exact unconditional	24	29	35	43	56
*Δ*=0.35					
McNemar asymptotic	13	15	18	23	29
McNemar asymptotic w/CC	18	21	24	28	34
McNemar exact	18	21	23	27	34
McNemar mid-*p*	15	17	20	24	30
Exact unconditional	15	17	20	24	29

^∗^Values above 100 estimated by linear extrapolation.

This table should not be used for sample size calculations.

#### The examples revisited

Table 6 presents the results of applying the five tests to the two examples introduced in the Background section. We have already observed that the asymptotic test and the exact conditional tests give quite different results for both examples. The asymptotic test with CC has *p*-values that are similar, but slightly higher, than the exact conditional test. The mid-*p* test and the exact unconditional test give results that largely agree with that of the asymptotic test. In both examples, the asymptotic, mid-*p*, and exact unconditional tests indicate stronger associations between airway hyper-responsiveness status and stem cell transplantation (Bentur et al. [1]) and between consolidation therapy and complete response (Cavo et al. [2]) than do the asymptotic test with CC and the exact conditional test. This difference in results is, perhaps, sufficiently great that different conclusions might be drawn. Because the asymptotic test with CC and the exact conditional test are highly conservative and have poor power, we do not recommend reporting the results of these two tests in any situation.

**Table 6:** ***P*-values of five tests using data from two published studies**

	**Bentur et al. **[1]	**Cavo et al. **[2]
	**2/21 vs 8/21**	**65/161 vs 75/161**
McNemar asymptotic	0.0339	0.0330
McNemar asymptotic w/CC	0.0771	0.0550
McNemar exact	0.0703	0.0525
McNemar mid-*p*	0.0391	0.0347
Exact unconditional	0.0353	0.0342

## Discussion

The evaluation study in this article revealed several interesting observations. First, that the conservatism of the McNemar exact conditional test can be severe. A large sample size is needed to bring its type I error rates above 3% for a 5% nominal significance level. Quite often, the type I error rates of the exact conditional test were half that of the nominal level or lower. A similar conservative behavior has been observed for other exact conditional methods, for instance, Fisher’s exact test for two independent binomial proportions [5] and the Cornfield exact confidence interval for the independent odds ratio [16]. This conservatism leads to poor power and a need for unnecessary large sample sizes. We do not recommend use of the McNemar exact conditional test in any situation.

Second, the McNemar mid-*p* test is a considerable improvement over the exact conditional test on which it is based. It performs almost at the same level as the exact unconditional test. Whereas the exact tests are guaranteed to have type I error rates bounded by the nominal level, no such claim can be made for the mid-*p* test. Nevertheless, the mid-*p* test did not violate the nominal level in any of the 9595 scenarios considered in this evaluation. For practical use, the mid-*p* test is at an advantage vis-a-vis the exact unconditional test. As shown in the supplementary materials, the mid-*p* test is readily calculated in many commonly used software packages, including the ubiquitous Excel. The exact unconditional test, on the other hand, is computationally complex and only available in StatXact (Cytel Inc.).

Third, the asymptotic McNemar test (without CC) performs surprisingly well, even for quite small sample sizes. It often violates the nominal significance level, but not by much. The largest type I error rate of the asymptotic McNemar test we observed in this study was 5.37% with a 5% nominal level. If that degree of infringement on the nominal level is acceptable, the asymptotic McNemar test is superior to the other tests. This is notably different from comparing two independent binomial proportions, where the asymptotic chi-squared test can produce substantial violations of the type I error rate in small samples [14].

The asymptotic test with CC performs similarly to—and sometimes even more conservatively than—the exact conditional test, and we do not recommend that it is used. This was expected, and is in line with the unequivocal recommendations against using the asymptotic chi-squared test with Yates’s CC for the analysis of the independent 2×2 table [5,13,17].

We have only evaluated tests based on the McNemar statistic. It is also possible to construct tests using the likelihood ratio statistic; however, Lloyd [18] found no practical difference between the two statistics. We prefer the much simpler—and widely used—McNemar statistic.

## Conclusions

The McNemar mid-*p* test is a considerably improvement on the McNemar exact conditional test. The mid-*p* test did not violate the nominal level in any of the 9595 scenarios considered in this article and is thus an excellent alternative to the vastly more complex exact unconditional test. The most powerful test is the McNemar asymptotic test (without CC), which we recommend if small but frequent violations of the nominal level is acceptable. We do not recommend use of the McNemar exact conditional test nor the asymptotic test with CC in any situation.

## Competing interests

The authors declare that they have no competing interests.

## Authors’ contributions

MWF conceived of the study, designed and carried out the evaluation of the tests, wrote an initial draft, and worked on the production of final draft. SL conceived of the study, participated in the design of the evaluation of the tests, and worked on the production of final draft. PL conceived of the study, participated in the design of the evaluation of the tests, and worked on the production of final draft. All authors read and approved the final manuscript.

## Pre-publication history

The pre-publication history for this paper can be accessed here:

http://www.biomedcentral.com/1471-2288/13/91/prepub

## Supplementary Material

Additional file 1 **How to calculate the McNemar mid-*P *test.** This document shows how to calculate the McNemar mid-*p* test using the software packages Excel, Matlab, R, SAS, SPSS, Stata, StatsDirect, and StatXact. Click here for file

Additional file 2 **Box-plots of type I error rates from the evaluation study.** This document shows box-plots of type I error rates from the total and various subregions of the evaluation study. Click here for file
