# Supplementary material for: The McNemar test for binary matched-pairs data: mid-p and asymptotic are better than exact conditional
Source: BMC Med Res Methodol. 2013 Jul 13;13:91. doi: 10.1186/1471-2288-13-91 (PMC3716987; doi:10.1186/1471-2288-13-91)
Supplement: Additional file 1 — How to calculate the McNemar mid-P test. This document shows how to calculate the McNemar mid-p test using the software packages Excel, Matlab, R, SAS, SPSS, Stata, StatsDirect, and StatXact. [file 1471-2288-13-91-S1.pdf]

How to calculate the McNemar mid-p test

(Supplementary materials)

Morten W. Fagerland Stian Lydersen Petter Laake

Introduction

To calculate the McNemar mid-p test, we start by calculating the McNemar exact conditional test. We condition on the number of discordant pairs $(n_{12} + n_{21})$, such that n_{12} is binomially distributed with parameters $n = n_{12} + n_{21}$ and $p = 0.5$. As shown in the main document, the one-sided exact conditional p-value equals the probability of at least n_{12} successes out of n binomial trials, and the two-sided p-value is twice the one-sided p-value:

$$\text{exact}\, p\text{-value} = 2 \cdot \texttt{binomcdf}(n_{12}, n, 0.5), \tag{1}$$

where `binomcdf()` is the binomial cumulative distribution function. Here, we have assumed—without loss of generality—that $n_{12} < n_{21}$. If $n_{12} = n_{21}$, the exact p-value equals 1.0.

We calculate the mid-p test by subtracting half the probability of the observed n_{12} from the exact one-sided p-value, then double it. For that we need the binomial probability density function, denoted by $\texttt{binompdf}(n_{12}, n, 0.5)$. The mid-$p$-value is thus

$$\begin{aligned} \text{mid-}p\text{-value} &= 2 \cdot \left[\texttt{binomcdf}(n_{12}, n, 0.5) - \frac{1}{2}\texttt{binompdf}(n_{12}, n, 0.5)\right] \\ &= \text{exact}\, p\text{-value} - \texttt{binompdf}(n_{12}, n, 0.5). \end{aligned} \tag{2}$$

If $n_{12} = n_{21}$, the mid-p-value equals $1 - 0.5 \cdot \texttt{binompdf}(n_{12}, n, 0.5)$.

The functions `binomcdf()` and `binompdf()` are readily obtained for most standard software packages. We illustrate the calculations of the mid-p test using data from the study by Bentur et al. [1] given in Table 1 in the main document, which we repeat here.

Table 1. Airway hyper-responsiveness (AHR) status before and after stem cell transplantation (SCT) in 21 children [1].

		After SCT		
		AHR	No AHR	Sum
Before SCT	AHR	1	1	2
	No AHR	7	12	19
	Sum	8	13	21

We have only checked the below calculations for the noted versions of the software packages and make no claim that the same approach is applicable to other versions.

1. Microsoft Excel (version 14)

The function `BINOM.DIST(x, n, p, cumulative)` returns both the point probability (`cumulative=0`) and the cumulative probability (`cumulative=1`) of the binomial distribution. Using the data in Table 6, the exact conditional p-value is obtained as `=2*BINOM.DIST(1; 8; 0,5; 1)`, and the mid-p-value is obtained as shown in the figure below. If $n_{12} = n_{21}$, the exact conditional p-value equals 1.0, and we obtain the mid-p-value using the expression `=1-BINOM.DIST(`n_{12}`;` n`; 0,5; 0)`.

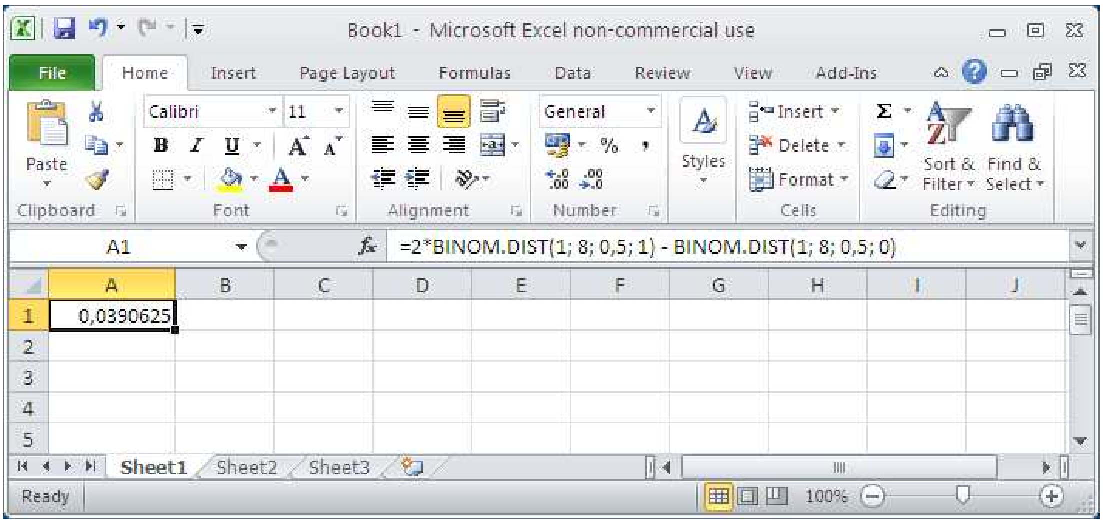

2. Matlab (version R2011b)

The two functions needed to calculate the mid-p test are available with the Statistics Toolbox add-on package. They are `binocdf(x, n, p)` and `binopdf(x, n, p)`. For the data in Table 6, we obtain the exact conditional and mid-p tests as shown below. When $n_{12} = n_{21}$, the exact conditional p-value equals 1.0, and the mid-p-value is calculated as `midp = 1 - 0.5*binopdf(`n_{12}`,` n`, .5)`.

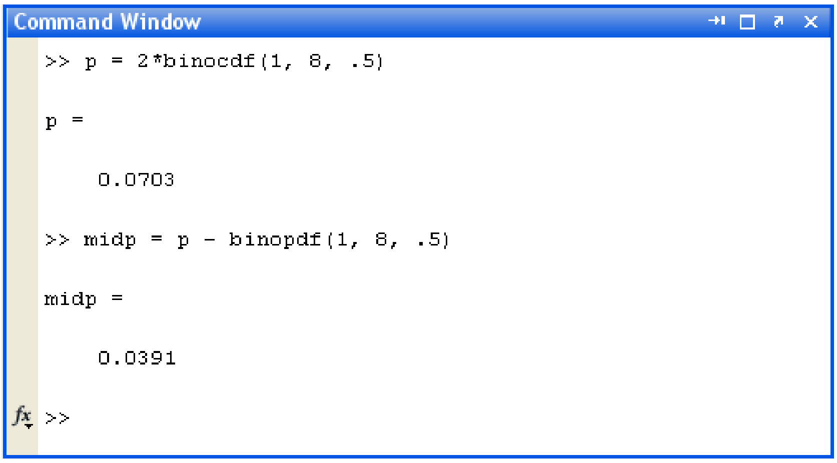

3. R (version 2.11.1)

`pbinom(x, n, p, lower.tail = TRUE)` and `dbinom(x, n, p)` compute the binomial cumulative distribution function and the binomial probability density function, respectively. The exact conditional and mid-p tests for the data in Table 6 can be calculated as shown below. If $n_{12} = n_{21}$, let the exact conditional p-value be 1.0 and compute the mid-p-value as `midp = 1 - 0.5*dbinom(`n_{12}`, `n`, 0.5)`.

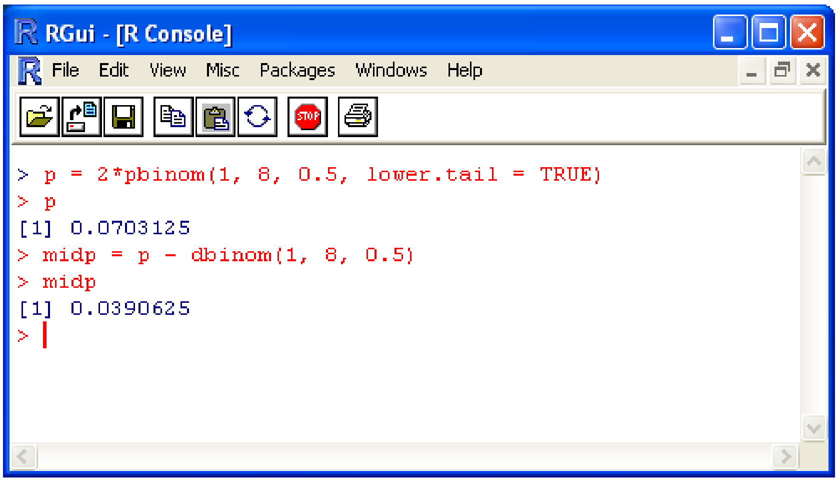

4. SAS (version 9.2)

The three figures below show how to calculate the exact conditional and asymptotic tests and the point probability of the observed table. The mid-p test is calculated as mid-p-value $= 0.0703 - 0.03125 = 0.0391$. If $n_{12} = n_{21}$, we obtain the mid-p-value as $1 - 0.5 \cdot$ `point_probability`.

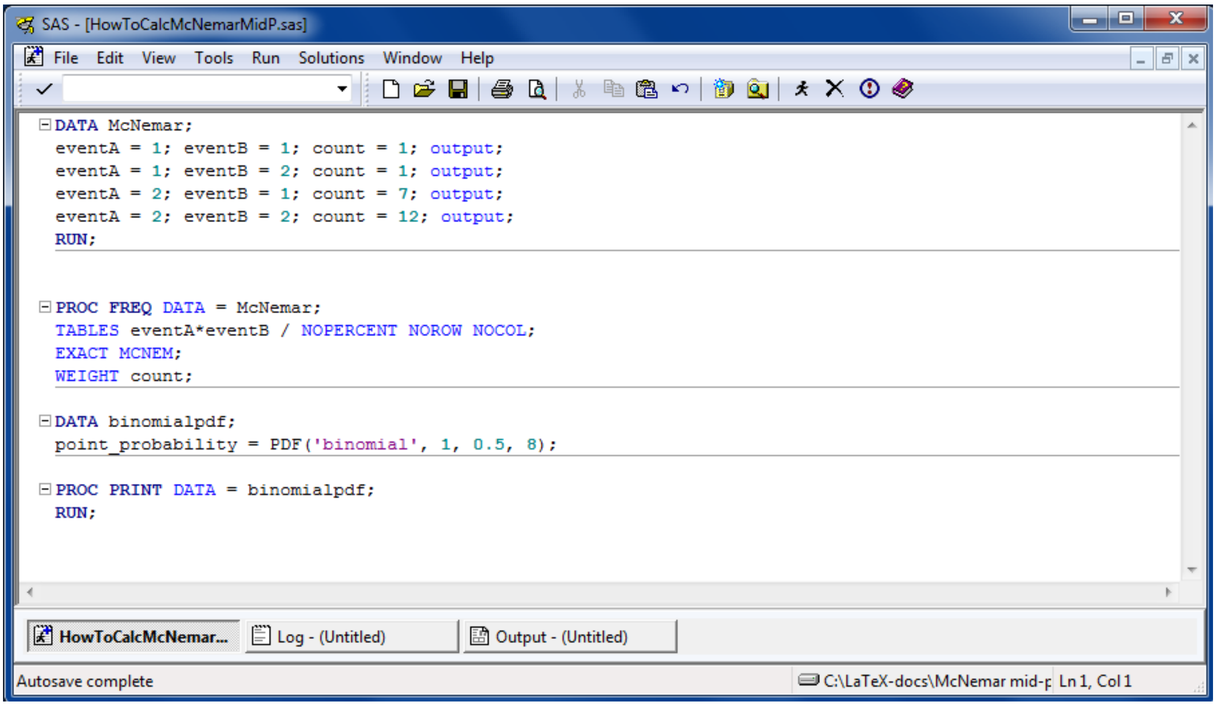

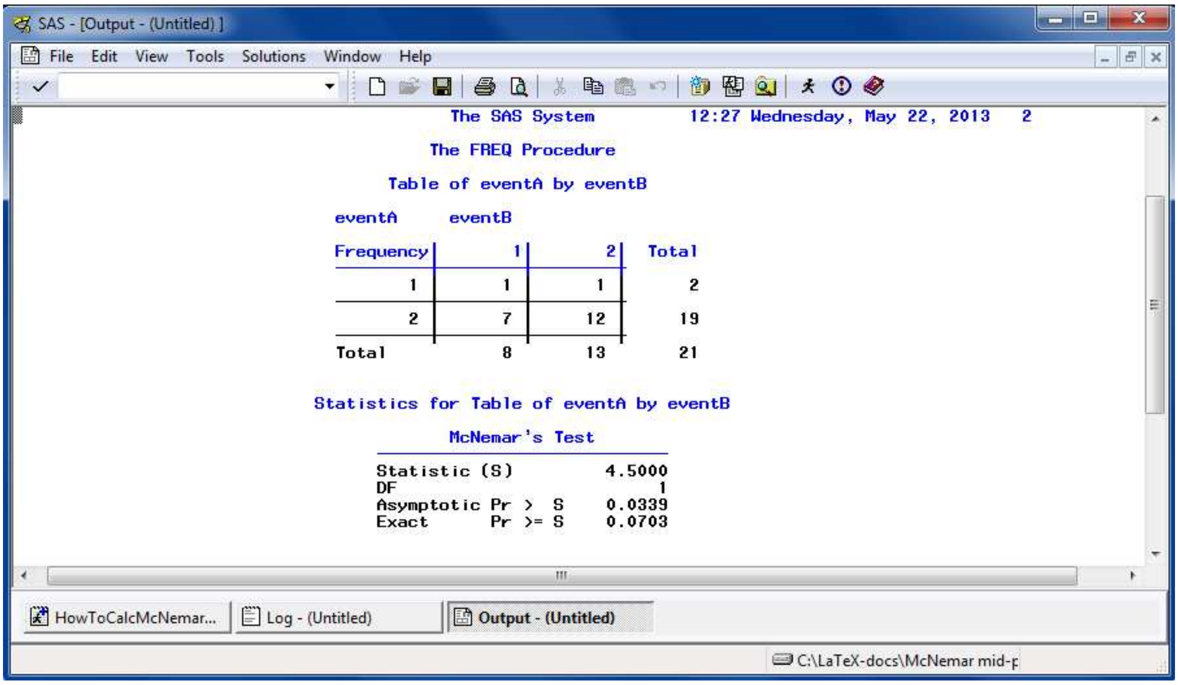
SAS - [Output - (Untitled)]
File Edit View Tools Solutions Window Help
The SAS System 12:27 Wednesday, May 22, 2013 2
The FREQ Procedure
Table of eventA by eventB
eventA eventB
Frequency 1 2 Total
1 1 1 2
2 7 12 19
Total 8 13 21
Statistics for Table of eventA by eventB
McNemar's Test
Statistic (S) 4.5000
DF 1
Asymptotic Pr > S 0.0339
Exact Pr >= S 0.0703
HowToCalcMcNemar...
Log - (Untitled)
Output - (Untitled)
C:\LaTeX-docs\McNemar mid-p

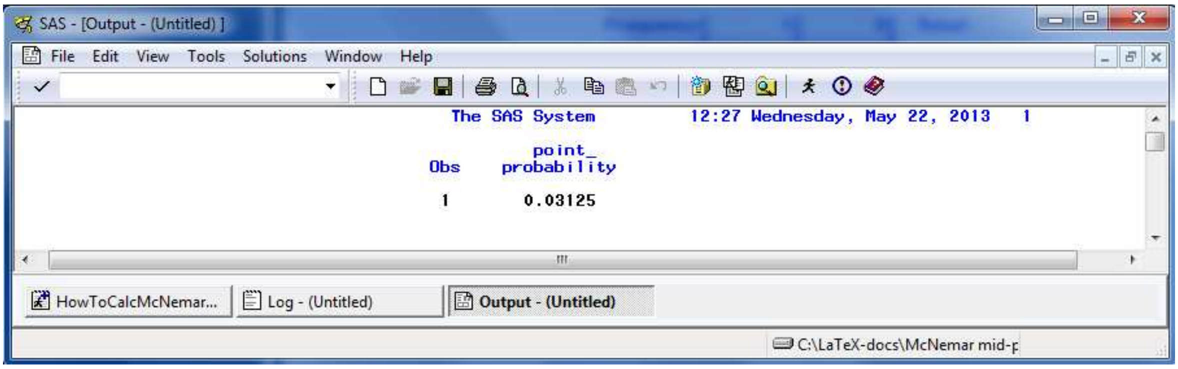
SAS - [Output - (Untitled)]
File Edit View Tools Solutions Window Help
The SAS System 12:27 Wednesday, May 22, 2013 1
Obs point_ probability
1 0.03125
HowToCalcMcNemar...
Log - (Untitled)
Output - (Untitled)
C:\LaTeX-docs\McNemar mid-p

5. IBM SPSS Statistics (version 20)

We use the function `Analyze -> Descriptive Statistics -> Crosstabs`. Put the appropriate variables in the boxes named “Row(s)” and “Column(s)” and press the “Exact” button. Select the “Exact” option and press “Continue”. Press the “Statistics” button and place a checkmark next to “McNemar”. Press “Continue”, then “OK”. The McNemar mid-p test is obtained by subtracting the value of “Point Probability” from “Exact Sig. (2-sided)”: mid-p-value $= 0.070 - 0.031 = 0.039$. When $n_{12} = n_{21}$, calculate the mid-p-value as $1 - 0.5 \cdot$ `Point Probability`.

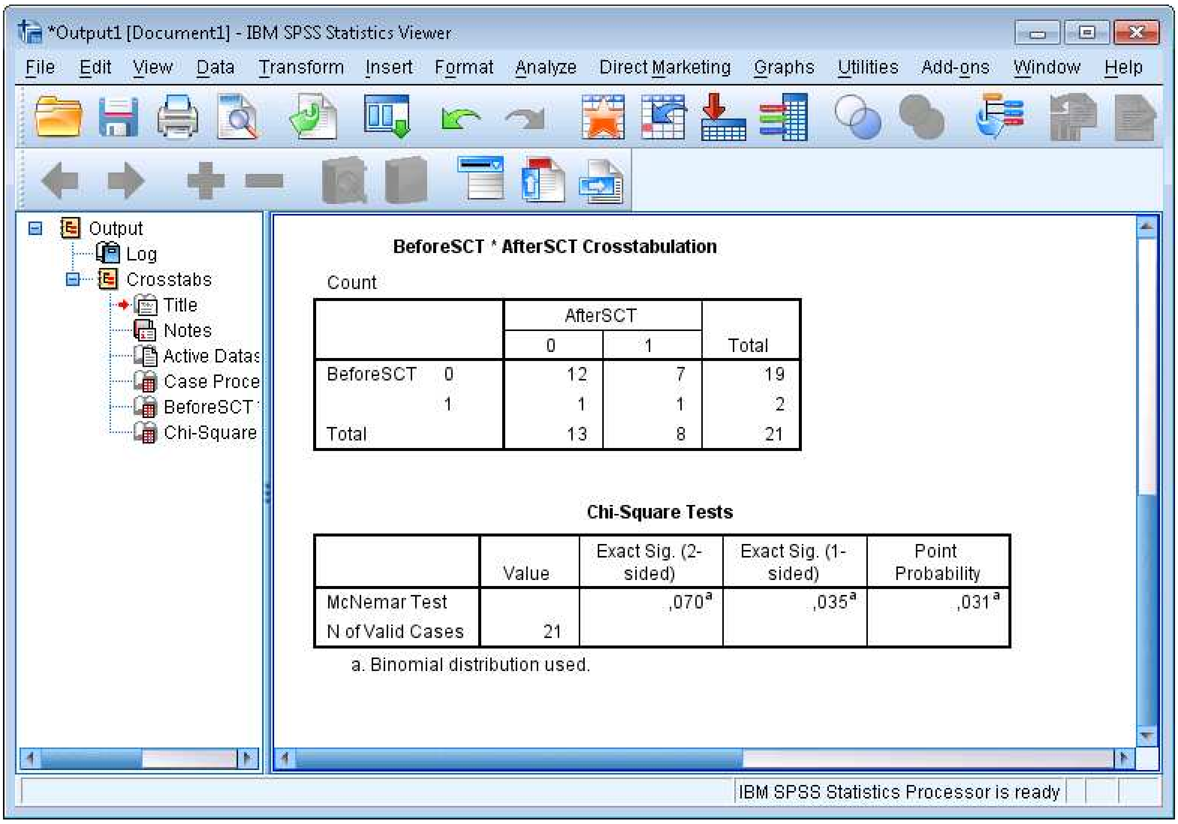

BeforeSCT * AfterSCT Crosstabulation

Count

		AfterSCT		Total
		0	1	
BeforeSCT	0	12	7	19
	1	1	1	2
Total		13	8	21

Chi-Square Tests

	Value	Exact Sig. (2-sided)	Exact Sig. (1-sided)	Point Probability
McNemar Test		,070[a]	,035[a]	,031[a]
N of Valid Cases	21			

a. Binomial distribution used.

6. Stata (version 12.1)

The exact conditional and asymptotic McNemar tests are obtained with the commands `mcc` and `mcci`, where the first command uses variables from the current dataset and the second command is a calculator version. Here, we show how to use the calculator version. The probability of the observed table is obtained using the function `binomialp(n, x, p)`. We calculate the mid-p test for the data in Table 6 as shown below. If $n_{12} = n_{21}$, we obtain the mid-p-value as `display 1 - 0.5*binomialp(`n`, `n_{12}`, .5)`.

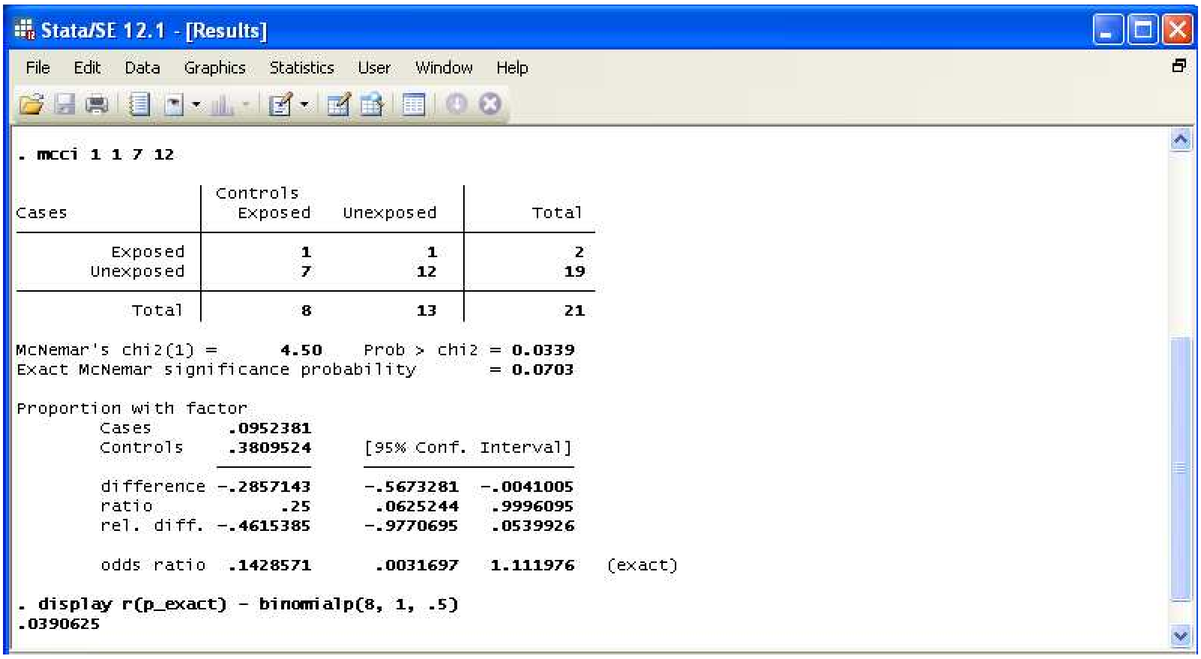


```
. mcci 1 1 7 12

                 | Controls               |
Cases            |   Exposed   Unexposed  |      Total
-----------------+------------------------+-----------
         Exposed |         1           1  |          2
       Unexposed |         7          12  |         19
-----------------+------------------------+-----------
           Total |         8          13  |         21

McNemar's chi2(1) =      4.50    Prob > chi2 = 0.0339
Exact McNemar significance probability       = 0.0703

Proportion with factor
        Cases       .0952381
        Controls    .3809524     [95% Conf. Interval]
                   ---------     --------------------
        difference -.2857143     -.5673281  -.0041005
        ratio            .25      .0625244   .9996095
        rel. diff. -.4615385     -.9770695   .0539926

        odds ratio  .1428571      .0031697   1.111976   (exact)

. display r(p_exact) - binomialp(8, 1, .5)
.0390625
```

7. StatsDirect (version 2.5.6)

In StatsDirect, both the exact conditional test and the mid-p test are given when using `Analysis -> Proportions -> Paired`. The p-value of the exact test is given as “Exact two sided P”, and the mid-p-value is given as “Exact two sided mid P”. When $n_{12} = n_{21}$, StatsDirect gives `Exact two sided P > 0,9999` and `Exact two sided mid P > 0,9999`.

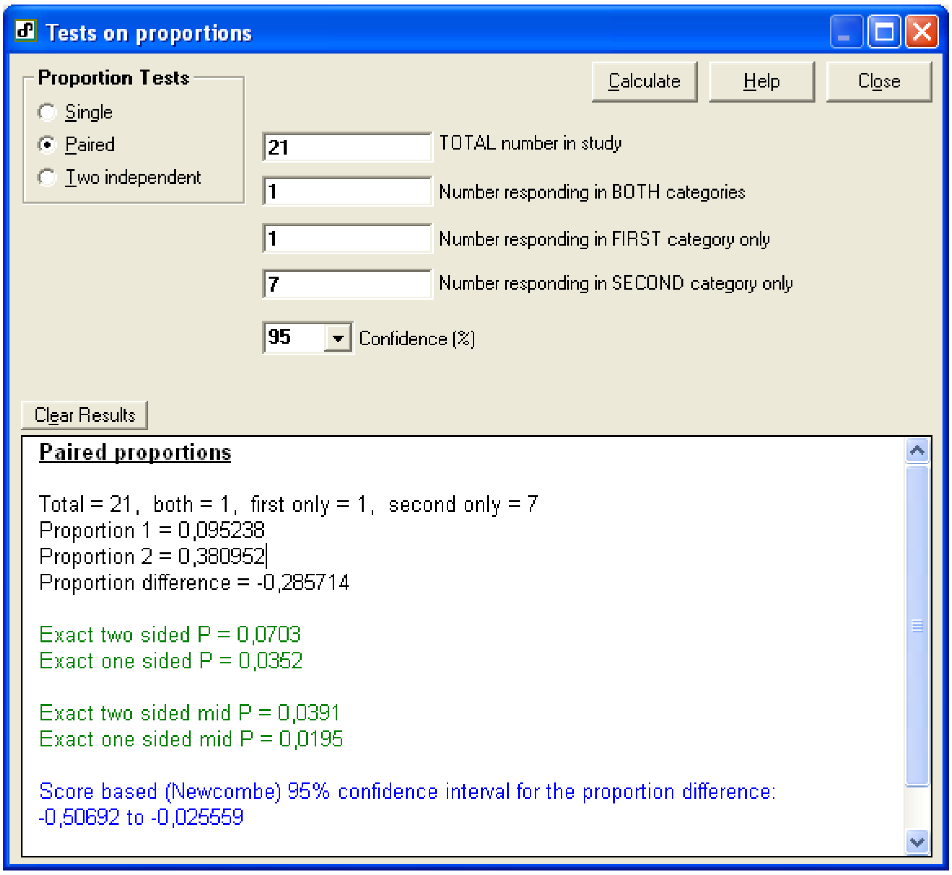

8. StatXact (version 9)

The p-value of the exact conditional test and the point probability of the observed table are obtained from `Nonparametrics -> Paired Samples -> McNemar`. In the "Inference" table, the p-value of the exact conditional test is given in the cell with row name "Exact (Conditional)" and column name "2*1-Sided". The point probability of the observed table is given in the cell with row name "Exact (Conditional)" and column name "Point Prob.", as shown below. The mid-p test can be calculated as mid-p-value $= 0.07031 - 0.03125 = 0.0391$. If $n_{12} = n_{21}$, calculate the mid-p-value as $1 - 0.5 \cdot$ `Point Prob.`.

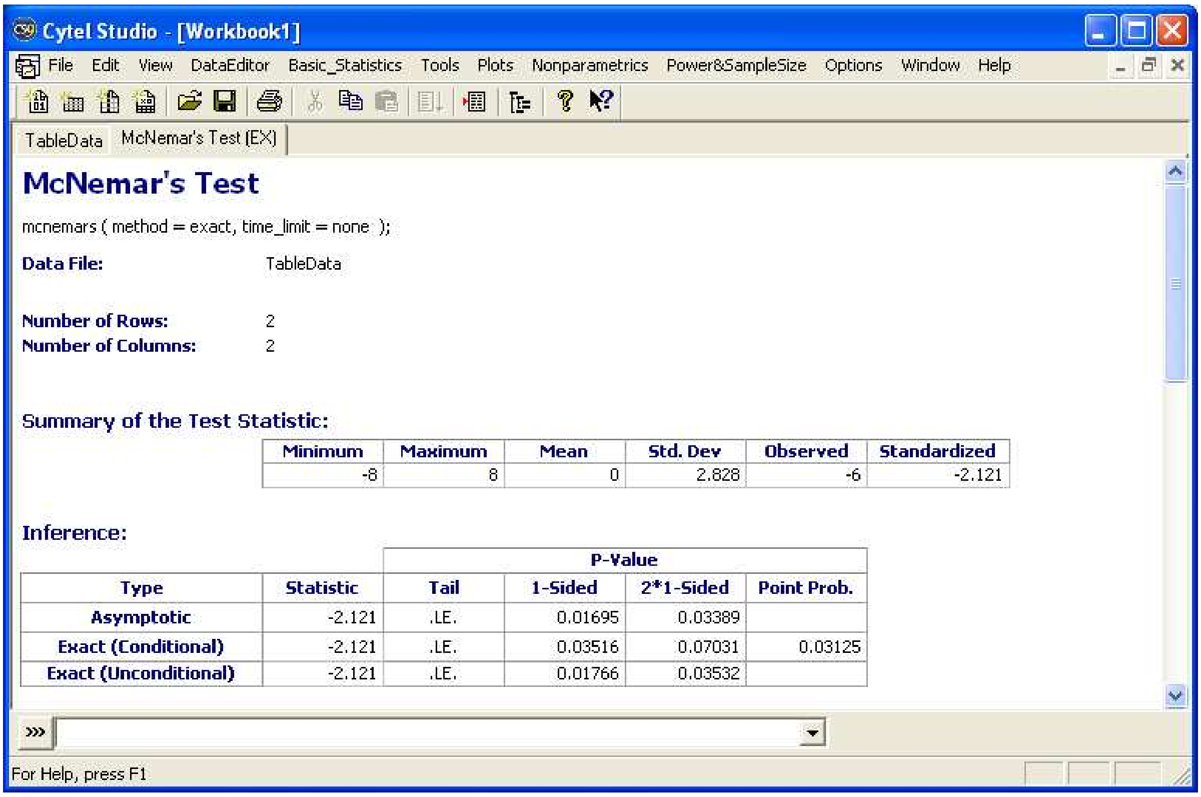

References

[1] Bentur L, Lapidot M, Livnat G, Hakim F, Lidroneta-Katz C, Porat I, Vilozni D, Elhasid R: **Airway reactivity in children before and after stem cell transplantation.** *Pediatr Pulm* 2009; **44**:845–850.
