# Supplementary material for: The McNemar test for binary matched-pairs data: mid-p and asymptotic are better than exact conditional
Source: BMC Med Res Methodol. 2013 Jul 13;13:91. doi: 10.1186/1471-2288-13-91 (PMC3716987; doi:10.1186/1471-2288-13-91)
Supplement: Additional file 2 — Box-plots of type I error rates from the evaluation study. This document shows box-plots of type I error rates from the total and various subregions of the evaluation study. [file 1471-2288-13-91-S2.pdf]

Box-plots of type I error rates from the evaluation study

(Supplementary materials II)

Morten W. Fagerland Stian Lydersen Petter Laake

Introduction

This supplementary materials document shows box-plots of type I error rates from the total and various subregions of the evaluation study, as defined in each figure caption.

The bottom and top of each box indicate the 25th and 75th percentiles, respectively, the central red line is the median, and the two whiskers (black dashed lines) extend to the most extreme data points not considered outliers. Outliers are plotted as red plus signs and defined as values that are larger than the 75th percentile $+ 1.5 \cdot \mathrm{IQR}$ or smaller than the 25th percentile $- 1.5 \cdot \mathrm{IQR}$, where IQR is the interquartile range (75th percentile $-$ 25th percentile).

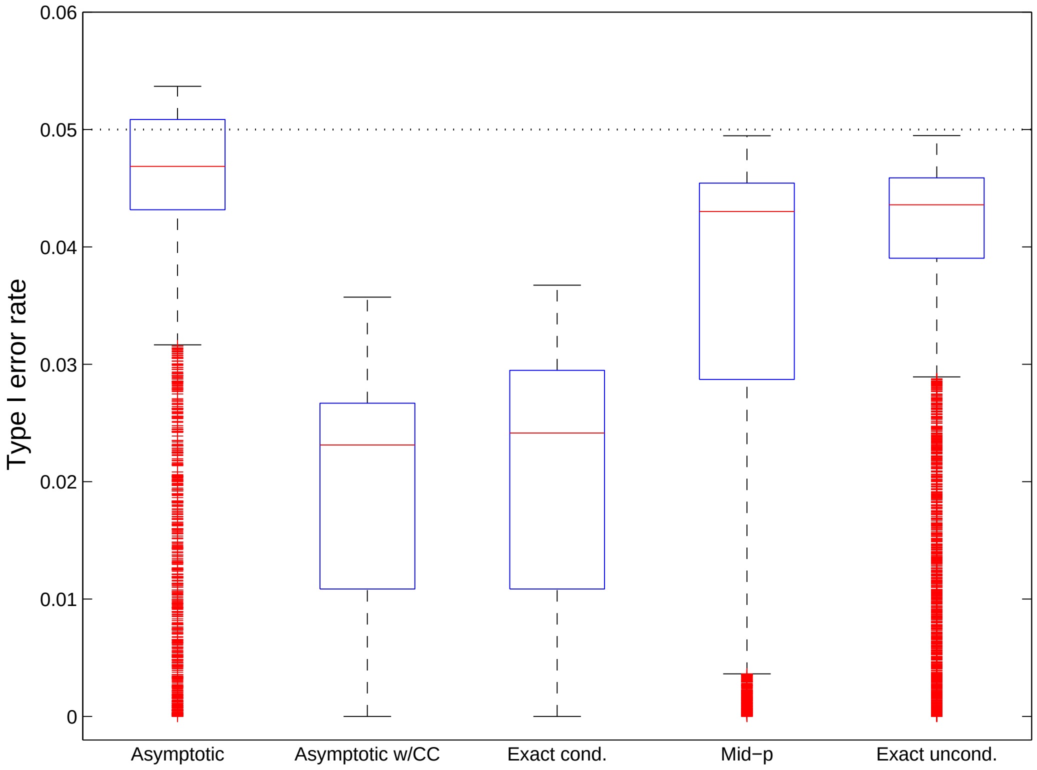

Figure 1: All 9595 scenarios.

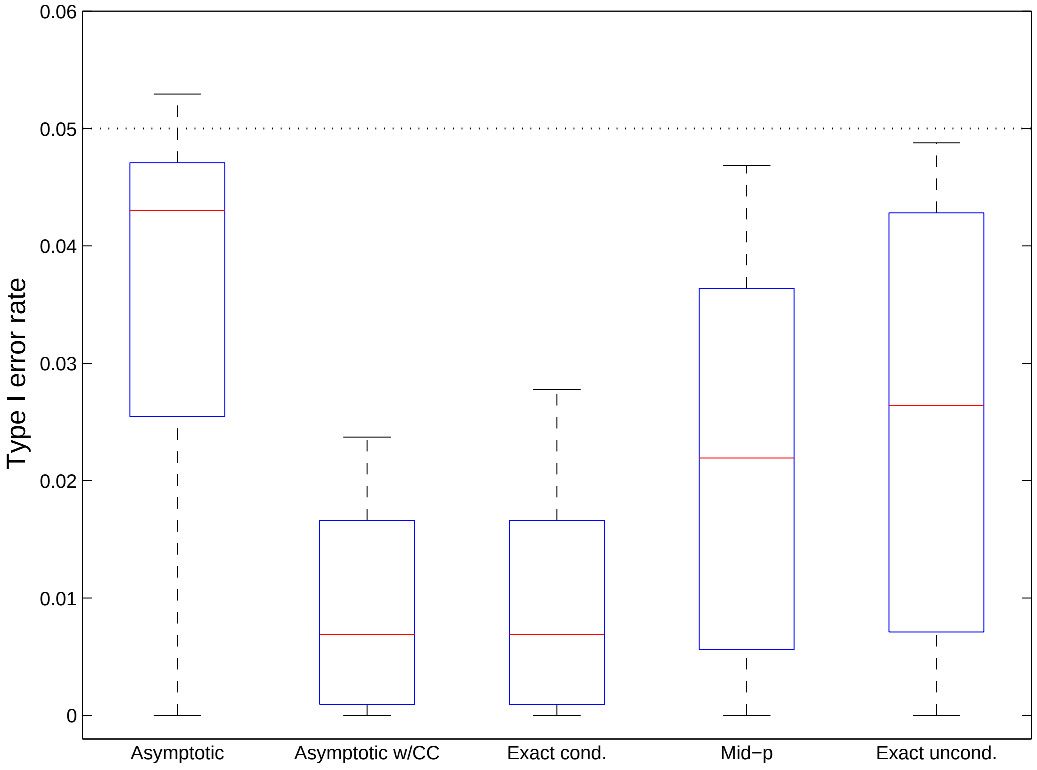

Figure 2: Subregion: $10 \leq N \leq 30$ (2525 scenarios).

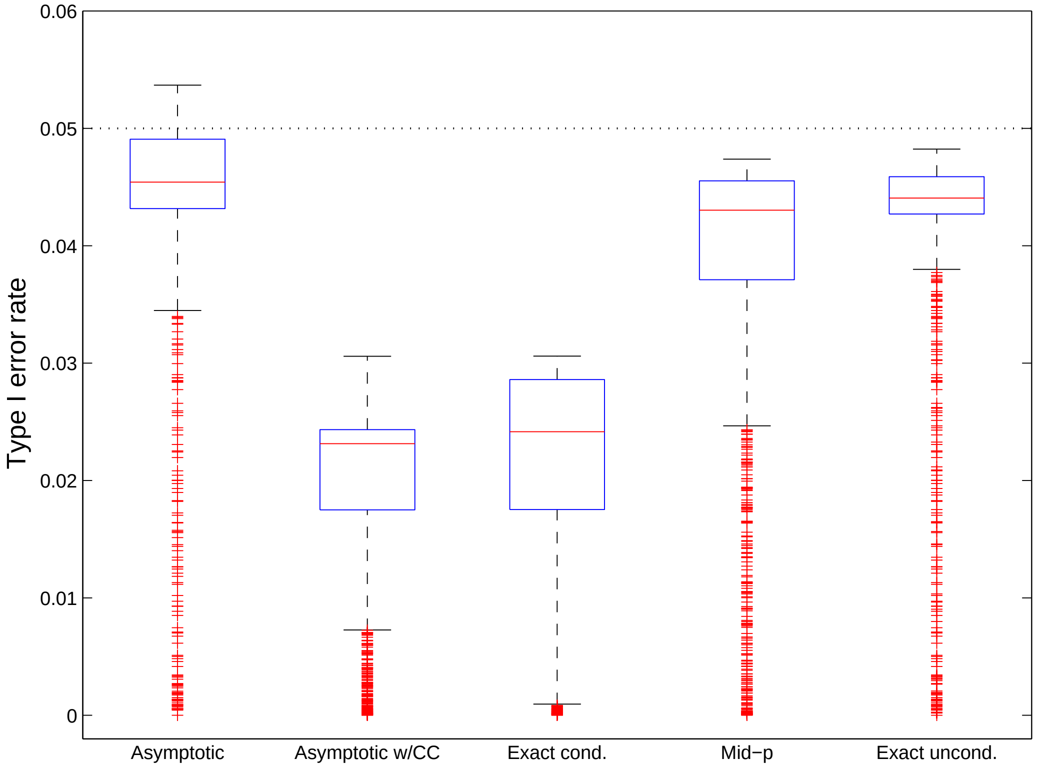

Figure 3: Subregion: $35 \leq N \leq 60$ (3030 scenarios).

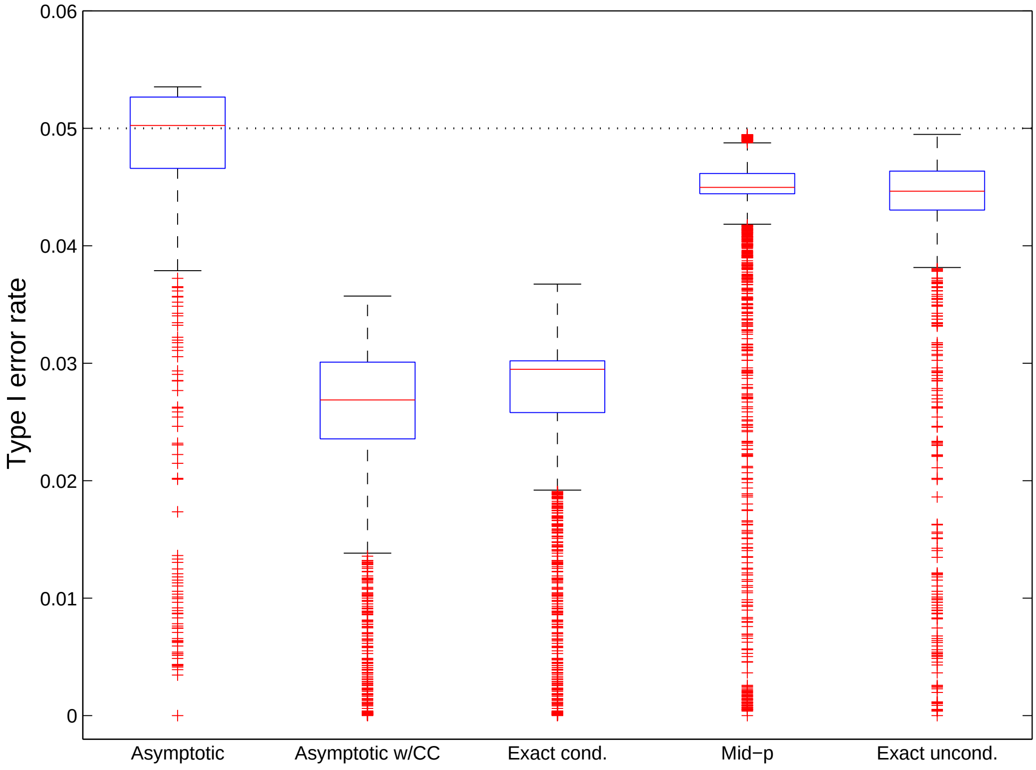

Figure 4: Subregion: $65 \leq N \leq 100$ (4040 scenarios).

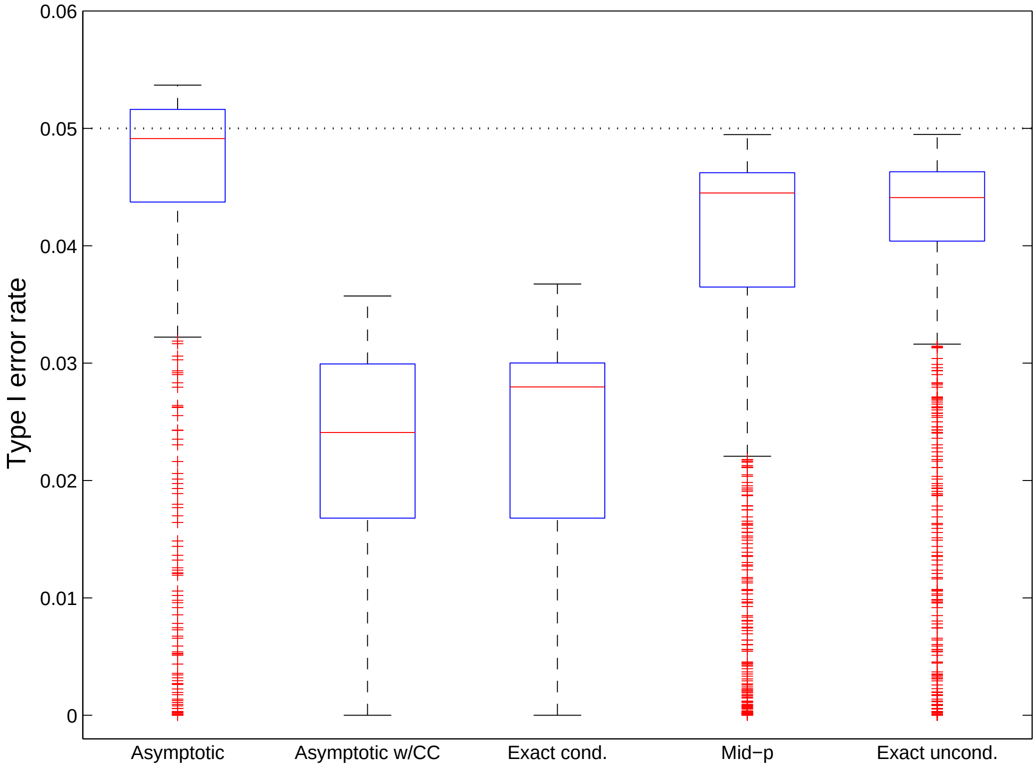

Figure 5: Subregion: $\theta = 1.0$ (1919 scenarios).

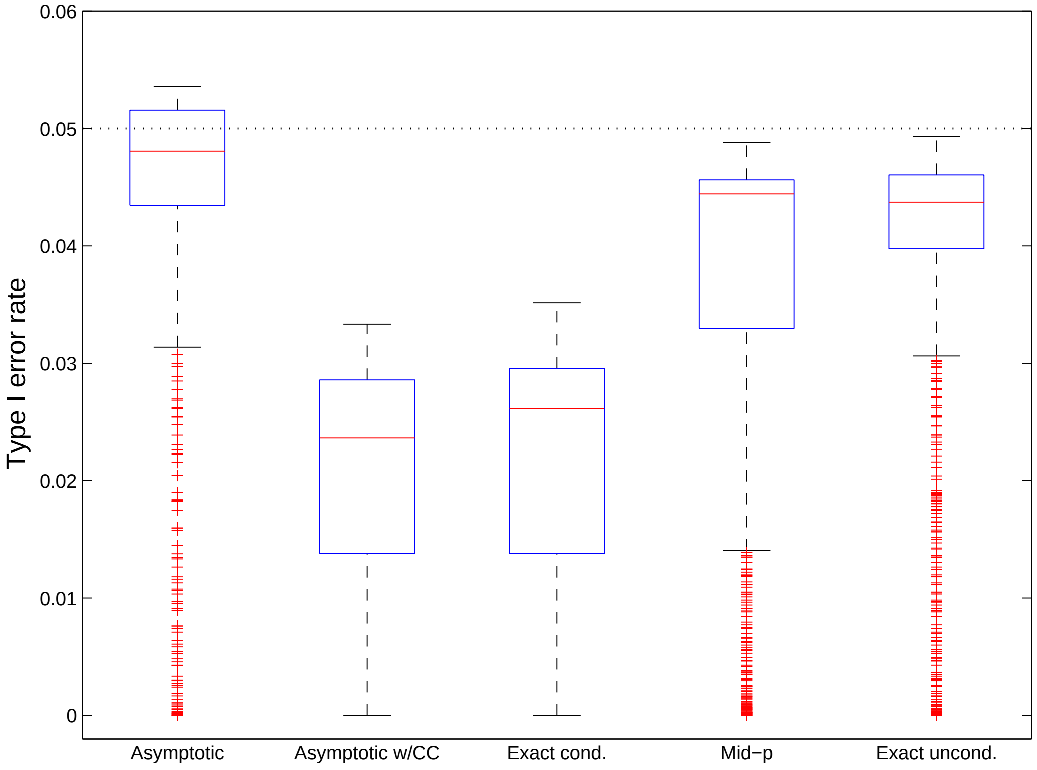

Figure 6: Subregion: $\theta = 2.0$ (1919 scenarios).

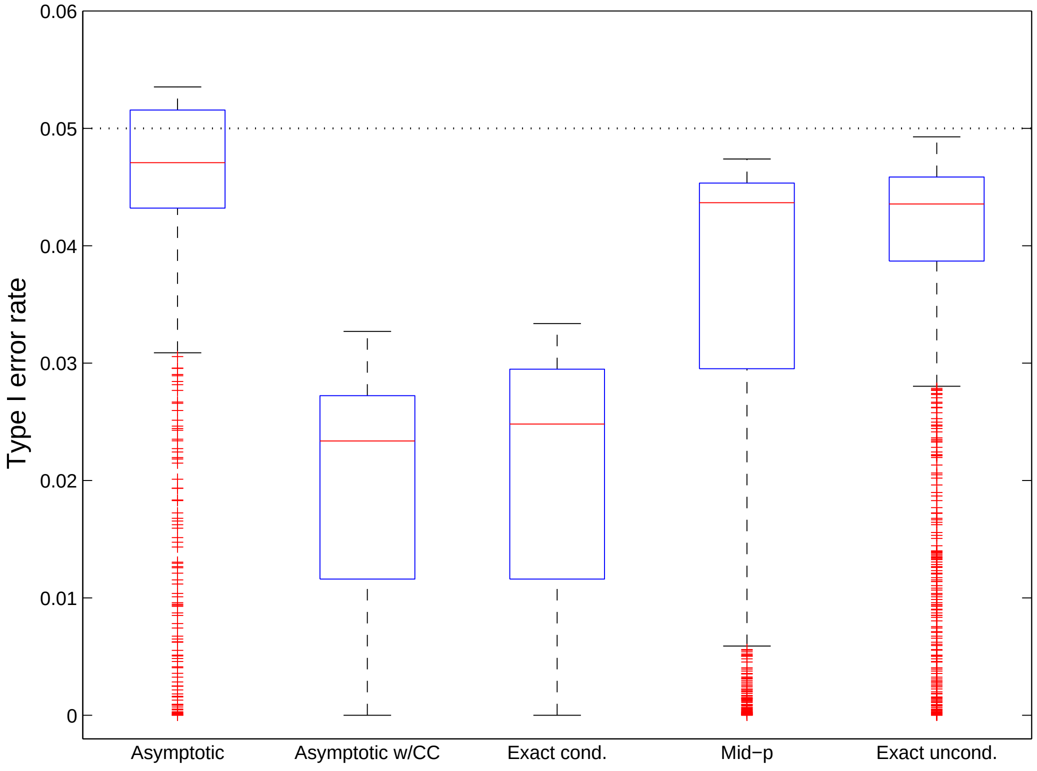

Figure 7: Subregion: $\theta = 3.0$ (1919 scenarios).

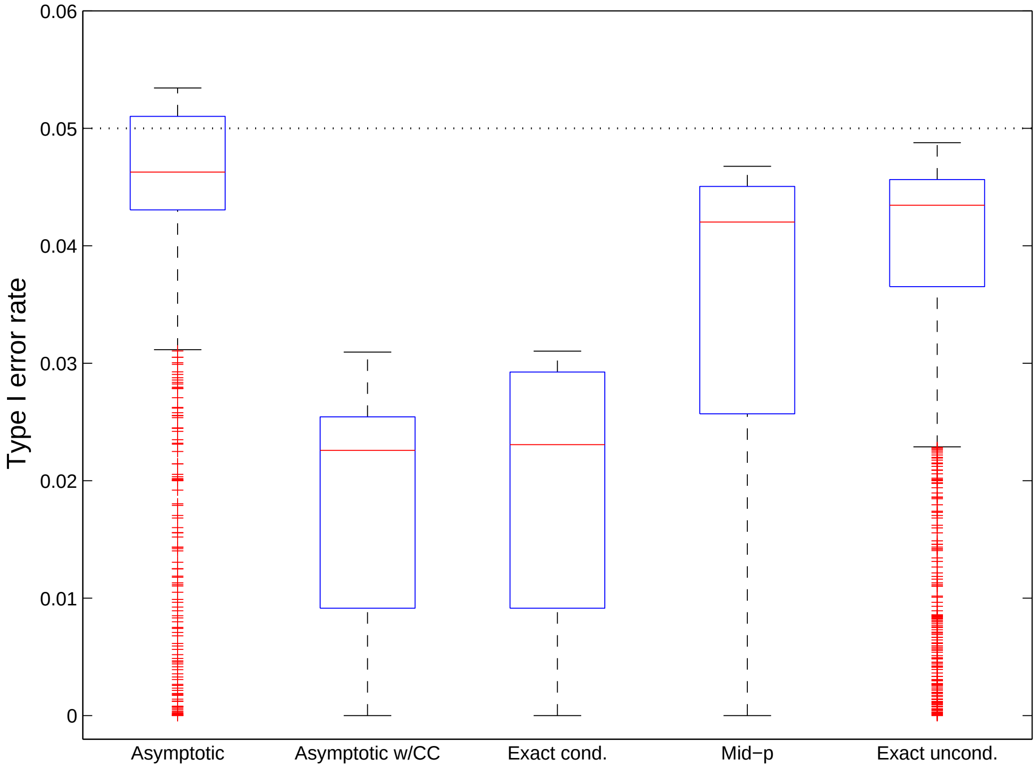

Figure 8: Subregion: $\theta = 5.0$ (1919 scenarios).

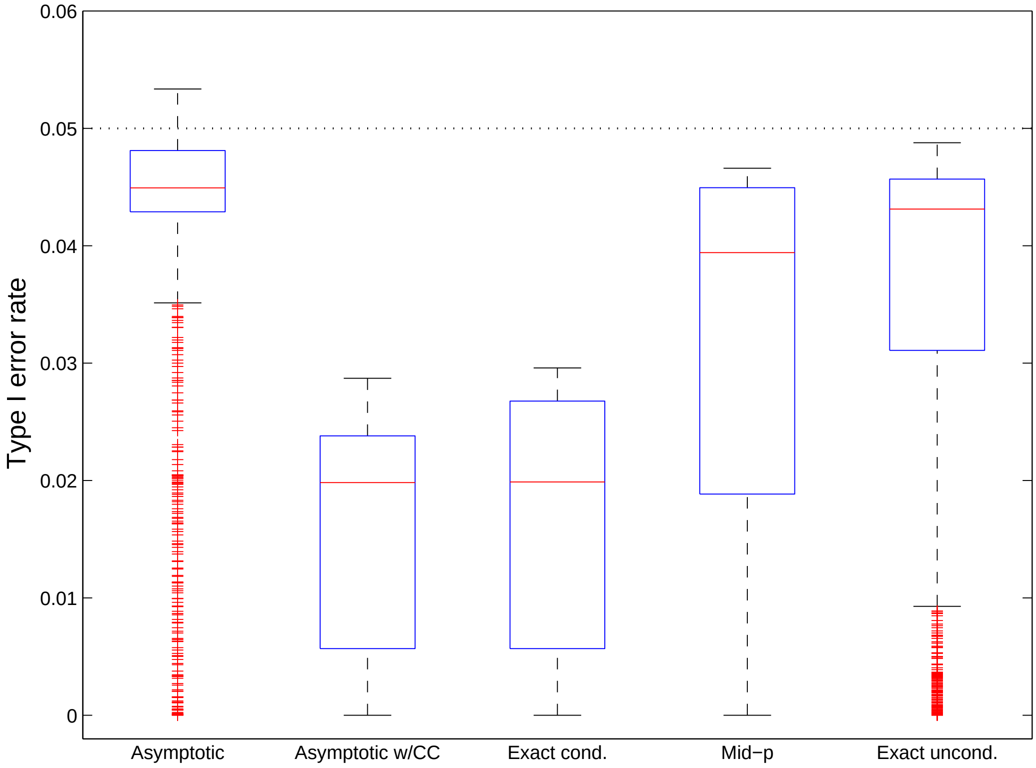

Figure 9: Subregion: $\theta = 10.0$ (1919 scenarios).

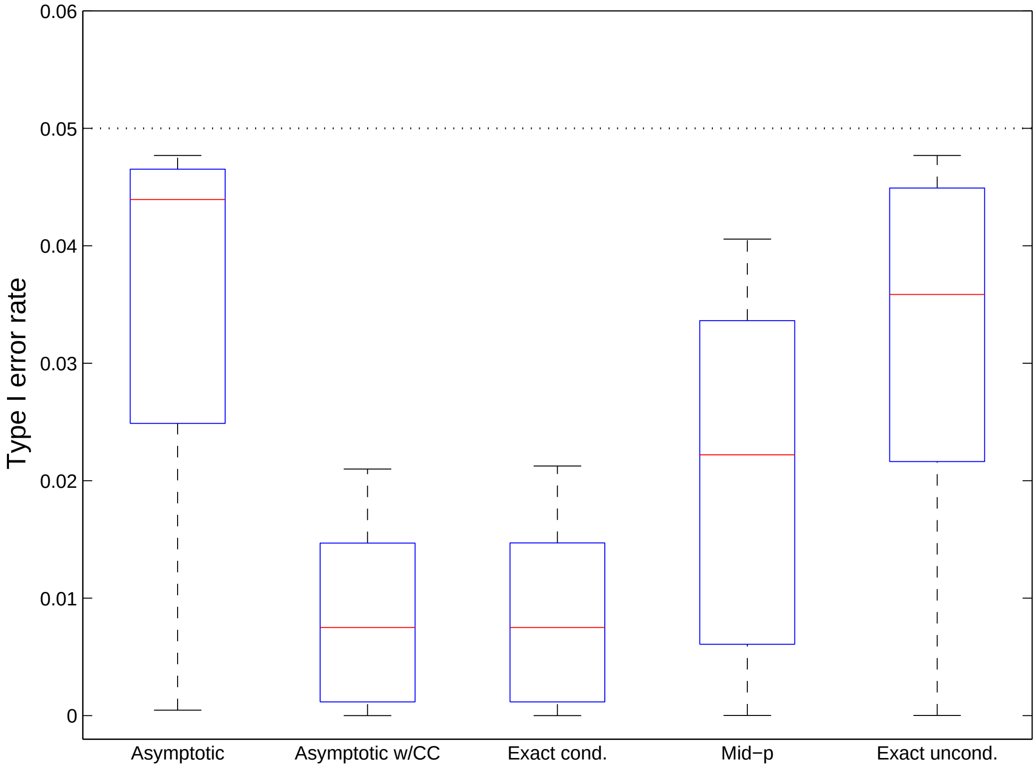

Figure 10: Subregion: $p_{1+} = p_{+1} = 0.05$ (95 scenarios).

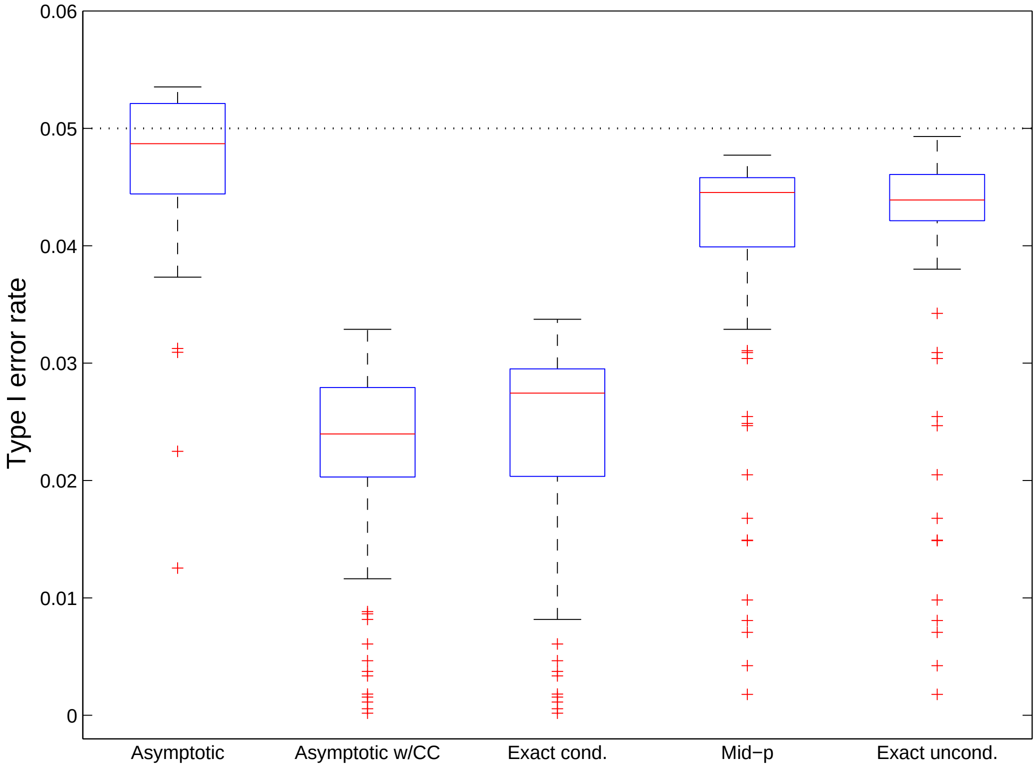

Figure 11: Subregion: $p_{1+} = p_{+1} = 0.25$ (95 scenarios).

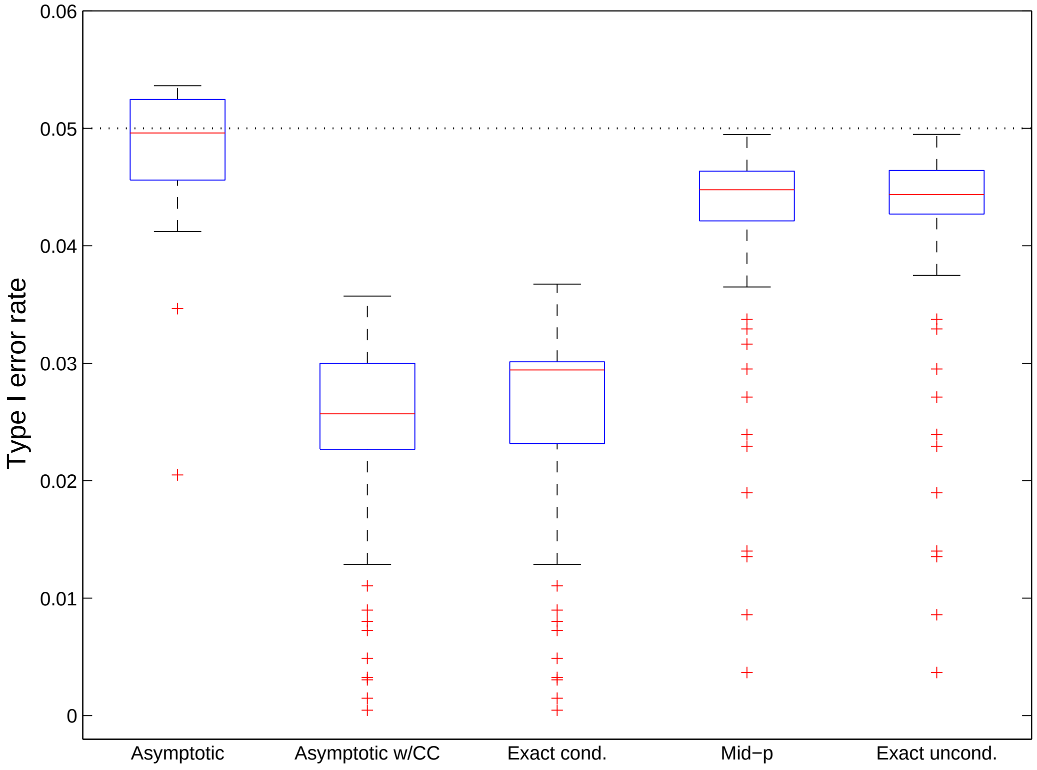

Figure 12: Subregion: $p_{1+} = p_{+1} = 0.50$ (95 scenarios).

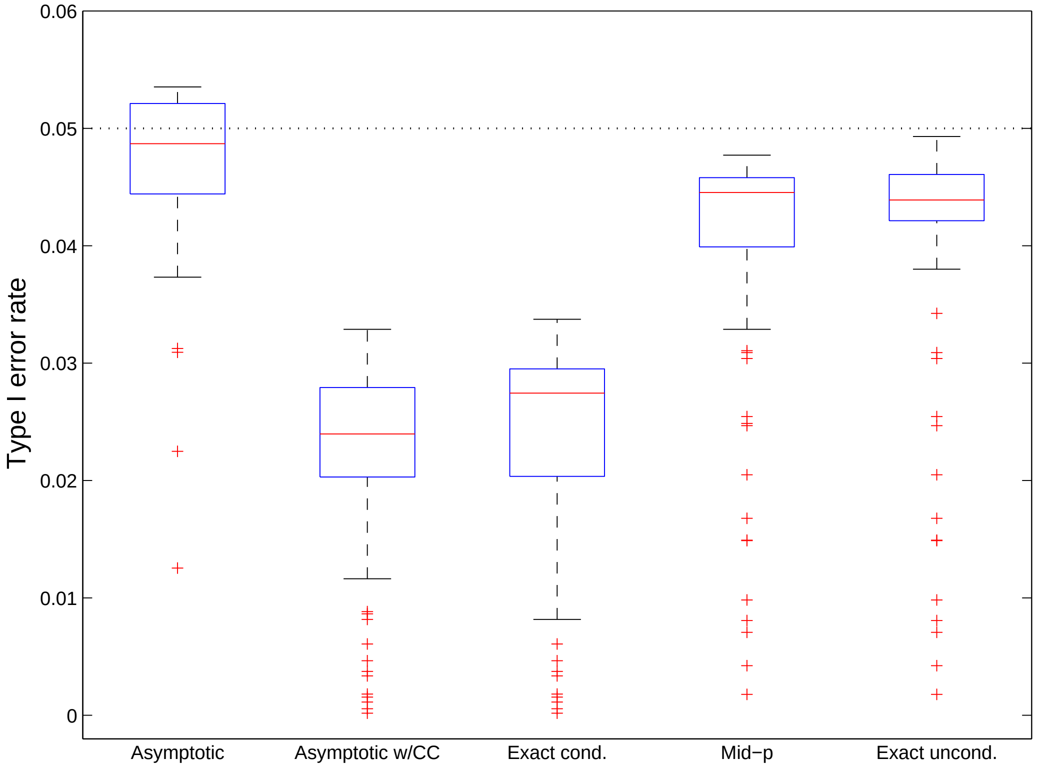

Figure 13: Subregion: $p_{1+} = p_{+1} = 0.75$ (95 scenarios).

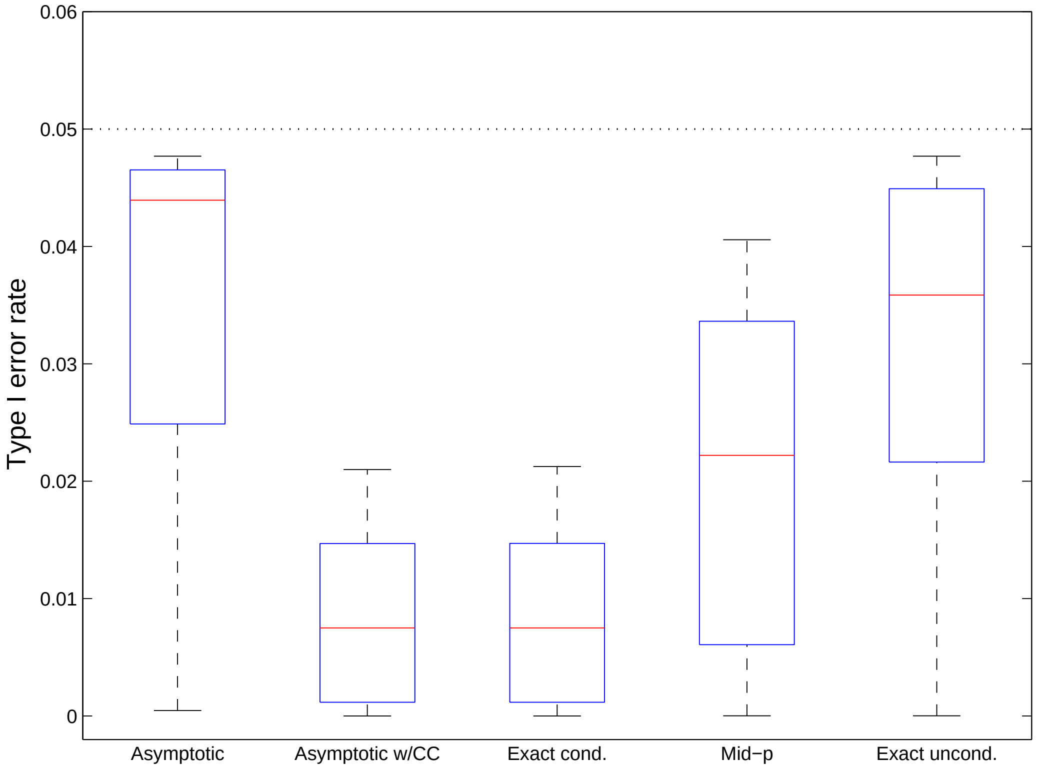

Figure 14: Subregion: $p_{1+} = p_{+1} = 0.95$ (95 scenarios).
